# Miscellaneous

**Published:** 1890-01

**Authors:** 


### MISCELLANEOUS.

Cure for a cold.—I have, two or three times within the last three months, been attacked by a violent cold in the head, the catarrh or discharge from nose and eyes being most distressing. On each occasion I have speedily cured myself by slicing two or three acid cooking-apples into a small saucepan of hot water, which I then boil for half an hour or so, stirring occasionally with a spoon until the apples were quite dissolved into a thin pulpy soup. This, sweetened with sugar, I then drank. In less than an hour afterwards I felt the cold giving way, and in two or three hours more it disappeared entirely. Not happening to have a lemon by me on the first occasion, I tried this remedy as a substitute, and can now confidently recommend it.—W. M.

### A RELIABLE INSURANCE.

E. C. Morris & Co., of Boston, Mass., had over fifty safes of their make in the great Lynn fire, which brought out their contents in fine shape, and since that time they have sold nearly 100 safes in that city alone. Over 100,000 of these safes are now in use, and hardly a day passes but what some of these are subjected to the severest tests, always bringing out their contents intact.

A safe is one of the most important things a business man purchases, and should be the very best. These safes are not high-priced for a first-class safe, but are very reasonable, and there is no reason why every one should not be provided with one.

### TOTAL DEPRAVITY.

This good old doctrine has of late been very much abused. Its inventor is entitled to our thanks for a term applicable to the printer or printer's devil, who was base enough to corrupt our contributor's lines, entitled "Bruno," in our December issue, by changing the plainly written word "divinely" in the last line, into "divinity." We are of opinion that those persons who have fallen away from the aforesaid doctrine, have never had much to do with printers, at least *some printers.* It is our intention to resign our place as chief of the grumbler's bureau or incase ourself in a full coat of mail, club proof. But after all, we cannot in justice blame "La Croix," for explaining to us some of the scientific rules of the ring. It wont go so hard with us when we get out of the hospital and have on our new bullet-proof suit. We trust our readers noted the blunder in "Bruno" and charged it to the right account, an account that in all likelihood never will be fully settled on this side of *Styx.*

### A POST-GRADUATE COURSE.

A wise, New Jersey mother, a widow, was in good circumstances, continuing a good business her husband had left her, and having four daughters, she gave to each of them the best education the city she lived in afforded. As it was the seat of a college, the schools were unusually good, and so was the society of the place. When the oldest daughter was graduated from school, her mother took her into the kitchen and initiated her into all the arts and mysteries of that department, and from that to up-stairs work, and to the providing the supplies—in short, to everything pertaining to house-keeping, even to presiding at the table. After she was thoroughly instructed in all this, and perfectly competent to do it, she and her mother took turns in having the entire charge of the house, a week about. When the other girls were graduated, they went in turn through the same course of instruction, and, consequently, at no time in their lives was house-keeping ever a bugbear to any of them.—*N. Y. Advocate.*

### THE LETTER.

(Sequel to lines in September issue.)

Laura Belle has written me
A very spicy letter.
She says she's yet content to be
A maid without the fetter,
But if I mean it fair—why she
Will watch for snowy weather,
And adds, alluding to my vow,
"It's snowing here a little now."

The fellows at the club are gay—
They pledge to me in bumpers,
Adopting Rip Van-Winkle's way
Of getting in their plumpers,
And threaten on the happy day
To raise a jolly rumpus!
The merry dogs are planning well,
But little wot of Laura Belle.

—BELKNAP.

